# Low-cost virtual instrumentation system of an energy-dispersive X-ray spectrometer for a scanning electron microscope

**DOI:** 10.1155/S1463924602000160

**Published:** 2002

**Authors:** Junfeng Lei, Libo Zeng, Ronggui Liu, Juntang Liu, Zelan Zhang, Jiming Hu

**Affiliations:** 1 Department of Analysis-Measurement Science Wuhan University Wuhan 430072 China; 2 College of Electronics Information Wuhan University Wuhan 430072 China

## Abstract

The paper describes an energy-dispersive X-ray spectrometer for a scanning electron microscope (SEM-EDXS). It was constructed using the new architecture of a virtual instrument (VI), which is low-cost, space-saving, fast and flexible way to develop the instrument. Computer-aided teaching (CAT) was used to develop the instrument and operation rather than a traditional instrument technique. The VI was designed using the object-oriented program language C++ and compact programmable logical devices (CPLD). These include spectra collection and processing, quantitative analysis and X-ray-intensity distribution analysis. The procedure is described in detail. The VI system gives an e¡ective and user-friendly human interface for the whole analytical task. Some examples are described.


Journal of Automated Methods & Management in Chemistry
Vol. 24, No. 4 (July–August 2002) pp. 121–125

### Low-cost virtual instrumentation system of an energy-dispersive X-ray spectrometer for a scanning electron microscope

**Junfeng Lei[1], Libo Zeng[2]*, Ronggui Liu[2], Juntang Liu[2], Zelan Zhang[2] and Jiming Hu[1]***

*[1] Department of Analysis-Measurement Science, Wuhan University, Wuhan 430072, PR China [2]College of Electronics Information, Wuhan University, Wuhan 430072, PR China*

*The paper describes an energy-dispersive X-ray spectrometer for a scanning electron microscope (SEM-EDXS). It was constructed using the new architecture of a virtual instrument (VI), which is low-cost, space-saving, fast and flexible way to develop the instrument. Computer-aided teaching (CAT) was used to develop the instrument and operation rather than a traditional instrument technique. The VI was designed using the object-oriented program language C++ and compact programmable logical devices (CPLD). These include spectra collection and processing, quantitative analysis and X-ray-intensity distribution analysis. The procedure is described in detail. The VI system gives an effective and user-friendly human interface for the whole analytical task. Some examples are described.*

### Introduction

Energy dispersive X-ray spectrometry (EDXS) is a fast, non-destructive and multi-elemental analysis technique. When it is connected to a scanning electron microscope (SEM), it can conveniently obtain the information of compositions with the surface characteristic of the sample. It is widely applied in research and in industry analysis, such as in material, interface and surface, mineralogy, etc. [1] Although the analytical technique is mature, the quantitative analysis technique can be improved especially for light elemental analysis, biological specimens and rough surfaces [2–4]. New computerized commercial instruments are expensive and lack the flexibility of a purpose-built function. The analysis speed of the traditional instrument is slow and the data processing is limited. It is difficult to link these with new methods of data processing because the installed computer cannot link to newer systems. The compromise between the cost and convenience is presented here.

The applications of virtual instrument (VI) have become more popular in modern instrument development and may play a role in avoiding this conflict. A VI in principle is a computer-based, software-driven instrument for testing, measuring and process control purposes. By incorporating standard computer hardware interfaces (such as plug-in board, RS-232 and IEEE 488) and using the computational power of a generic central processing unit (CPU), a VI defines its specific functions through software programming [5].

* To whom correspondence should be addressed. e-mail: jmhu@whu.edu.cn

Compared with hardware-based instruments, the advantage of a VI is its flexibility. This is of particular value in open-ended research studies. The functionality and user–machine interface of conventional test instruments are manufacturer-defined. Consequently, end-users have limited options to expand or modify the exiting functions. In contrast, the functionality of a VI can be explicitly defined, modified and expanded by its users through software programming.

In this work, the method is adopted with an eye to the future. VIs were written in C++, which performs all the analytical functionalities of SEM-EDXS. A complex programmable logical device (CPLD) was used to develop the hardware.

### Type of analysis [1]

The main functions of instrument include X-ray point analysis (XRPA); X-ray line scans (XRLS) and X-ray distribution images (XRDI).

XRPA is the basic functionality of SEM-EDXS, which can give the qualitative and quantitative analysis for a point (microregion) on the surface of the sample. Data collection includes manual, timing, counting and dead time correction. In general, the last three modes are mainly used for quantitative analysis with a standard sample while all the modes can be used for non-standard analysis.

The second analysis is XRLS. In this case, the electron beam is scanned slowly along a single line in the electron image of sample. The intensity for the element being investigated is plotted directly to the SEM or PC monitor. The X-ray line scans in the SEM can only show the relating variations of one elemental concentration, but it can give more information on PC.

The third mode is XRDI, which can calculate the approximate composition distribution of the surface of the sample. This analysis also can be fulfilled on the SEM or the PC using similar line scans. The analysis on the SEM will give the result of one element, while the PC can show the distribution of many elements.

### Hardware description

Figure 1 shows the EDXS hardware system configuration developed. This hardware includes the following modules: signal preprocessing, spectra pulse processing, line-

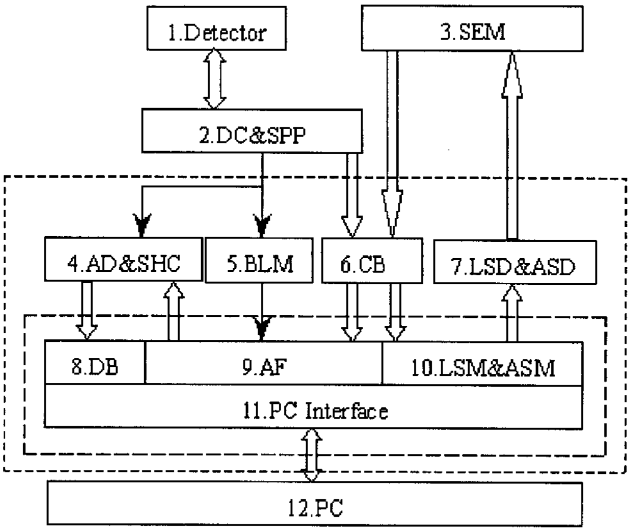

*Figure 1. Hardware modules diagram. (1) Si(Li) detector; (2) detector control and signal preprocessing module; (3) SEM; (4) A/D covert and sample/hold section; (5) baseline monitor; (6) control signal buffer; module (7) is the line-scanning and area-scanning drivers; (8) spectra data buffer; (9) analytical functionality; (10) line-scanning and area-scanning data buffer; (11) PC interface; (12) personal computer.*

scanning feedback and area-scanning feedback. The detector collects X-ray photons, and the photon is converted to an electric charge, which is proportional to the energy of the incident X-ray photon.

An EDAX9100 is used (EDAX Inc., 91 McKee Drive, Mahwah, NJ 07430, USA). The signal-processing circuit and the detector-controlling circuits for signal processing were designed in-house. The output signals of module 2 are a composite of the spectra pulse, the inhibited signal and the signal of the X-photon number. The spectra pulse is similar to a Gaussian function, and the band width of the pulse is about 80 μs. The peak-to-peak value is in the range +10 to −10 V. The inhibited signal comes from the detector discharges. When the signal is active, the whole system must stop working. The width of this signal is about 200 μs and the amplitude is 5 V. The third signal represents the number of X-photons, which is a 0.5-μs-wide digital pulse. One pulse represents an X-photon and the amplitude is 5 V. In figure 1 the two control signals are represented by the box arrow pointed to module 6, and the spectral pulse signal is the arrows directed to the modules 4 and 5. The long dashes box represents the on-chip functionalities, which mainly include some control facilities and the computer interface. These are implemented in two electrically reprogrammable logic devices, the EPM7128S (Altera Digital Library; Altera Corp., 1998, 101 Innovation Drive, San Jose, CA 95134, USA) with an in-system programmability (ISP) technique that provides a flexible means of upgrading the instrument or changing its functionality. The short dashes box represents on-board realization, mainly including some interface such as A/D, S/H and D/A. The AD IC is a 12-bit and 10-μs device. For the 80-μs pulse width, it is fast enough to data transform and transmit to computer without the loss of the pulse. The main advantage of the development is the instrument control, and functions can integrate together into a single board, even into a single chip. It also decreases the space taken by the instrument. In addition, the integrated realization may reduce the faulty ratio of the instrument.

The multichannel analyser (MCA) is implemented by software. A fast AD method is used for the digitalized pulse peak. Because the width of the pulse time is larger than the sum of the AD convert time and the computer IO operation time, the computer memory becomes the memory of the MCA. This method of implementation provides a flexible way for data handling and displaying the data and it reduces the complexity of the hardware implementation.

A more in-depth description of the section is included in Junfeng Lei *et al.* [6].

### Software architecture

The whole programme, which can run under win9x on any IBM-compatible personal computer, was written in C++. The instrument's operation interface uses a combination of menus and toolbars. The hardware control and operation was encapsulated in a Virtual Device Driver (VxD) programme developed only for win9x.

The programme for the work modules of the whole system are described below. The workflow of the analytical modules is shown in figure 2. A more detailed description of the section is included in Libo Zeng *et al.* [7].

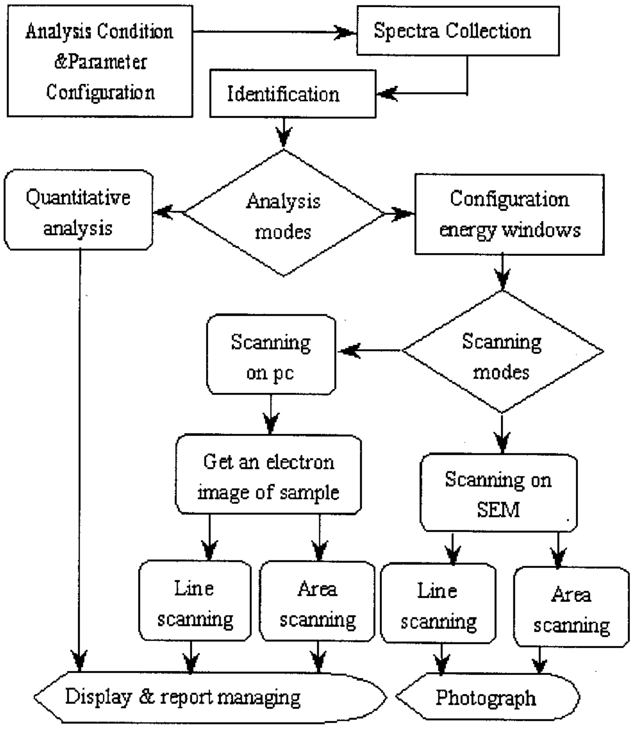

*Figure 2. Flow diagram of the X-ray energy-dispersive analytical instrument.*

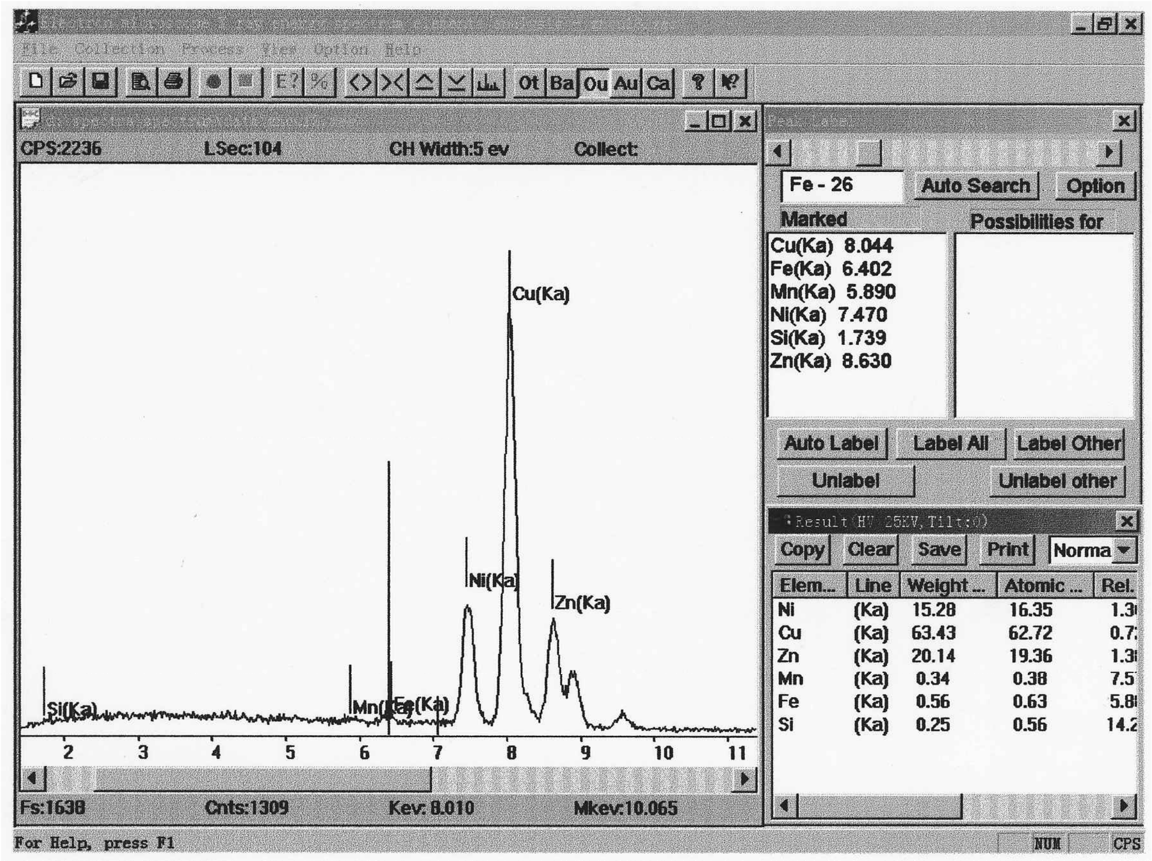

*Figure 3. Interface of a quantitative analysis of a standard sample (Zn/Cu alloy: GBW02104).*

*Calibration*

Because of the drift of the instrument circuit, the instrument often needs to correct the energy gauge. The instrument supplies hardware and software calibration for this task. The hardware method is applied only when the instrument is initially installed or the change of the energy gauge is large, while the software method adopts an algorithm such that the energy gauge is dilated and shrunk and translated linearly. This method is often used for user scaling for a small change of energy gauge and it is enough to handle the majority of cases.

*Spectra collection and identification*

The spectra collection adopted a soft MCA. The spectra pulse discrimination was fulfilled by the hardware, but the classification of the spectra pulses in terms of the pulse height and run-time display were provided by a computer program.

The element identification often used is the look-up table. Because of the diversity of the sample and the complexity of the spectra, the complete automation of the identification is completely reliable. In this system, identification is provided by a semi-automated identification assisted by user intervention.

*X-ray point analysis*

To obtain the composition information of a sample, the system supplies quantitative or semiquantitative analysis using the ZAF correction algorithm similar to Frame C. There are three methods for background subtraction available to user in the program: interpolation, background simulation and background filter. For each different spectrum character of the different samples, these methods supply different selection criteria for background subtraction in individual analytical cases.

Spectra stripping can be fulfilled by peak fitting of computer-generated peaks whose profile is a modified Gaussian function or by library spectra if the spectra overlap is acceptable. In addition, miscellaneous peaks are automatically removed from the spectrum by a menu command for all identified elements.

As mentioned above, there are many kinds of algorithms for quantitative analysis that are applicable for all cases. It is important that the original spectra data are open for users. In this system, the data are stored in an ASCII file. The spectra data are 4096 double-word arrays at the front of the file as well as data for display of the spectra.

*Intensity distribution analysis*

This mode can only supply qualitative information of sample compositions in the analytical region because the topograph of the sample seriously influences the accuracy of analytical mode. It includes four approaches to get the intensity distribution: XRLS on the SEM and computer, and XRDI on the SEM and computer.

The key implementation of the function is the corresponding relation between electrons' image and the intensity of the X-ray along the analytical line or the whole

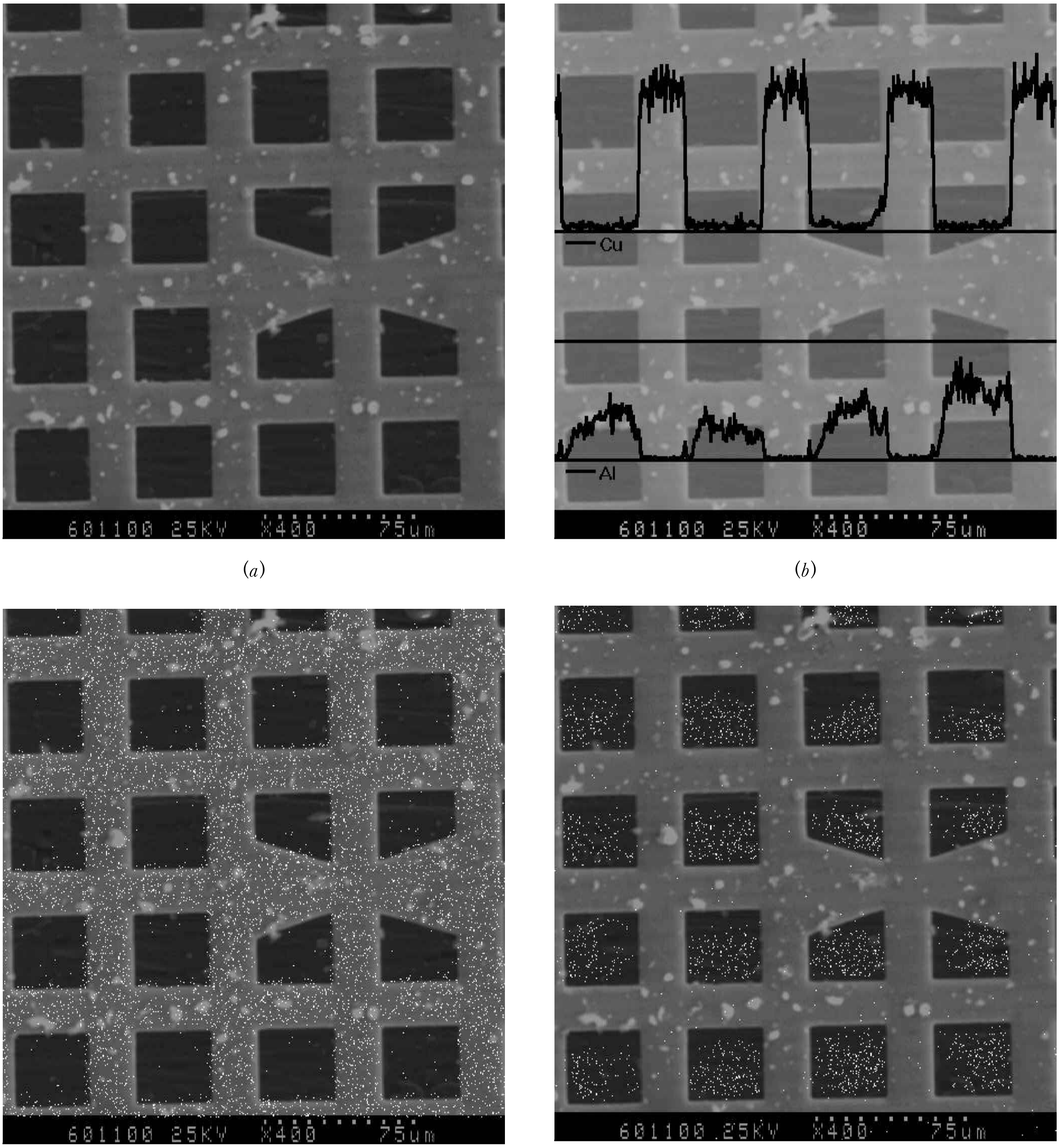

*Figure 4. Quasidigital mapping of X-ray intensity on a personal computer. The specimen is a Cu net on a sample holder whose composition is Al. (a) Secondary electrons image of the specimen. (b) Line-scanning of two elementals (Cu/Al). The curve is the distribution of Cu and Al; the straight line in the image is the position of line-scanning on the specimen. (c) Area-scanning of Cu. (d) Area-scanning of Al. The white dots in (c) and (d) represent the intensity distribution of the character X-ray of Cu and Al on the surface of the specimen.*

SEM image. Two parameters are supplied for the XRLS to adjust the corresponding relationship between the intensity distribution curve and the scanning line. Two other parameters are used to enhance the effect of the display at the first installation, while the adjustment of only one parameter is needed to enhance the display effect within general analysis. For XRDI, the expression of intensity distribution is simpler than XRLS, so the two parameters only need to adjust the corresponding relation between the intensity distribution and the image. A dialogue is supplied to set up the parameters, and an empirical expression is adopted to adjust the algorithm

*Table 1. Results of the quantitative analysis of a standard sample (Zn/Cu alloy: GBW02104).*

| Elemental | Nominal (WT%) | This work (WT%) | Relative err.% |
|---|---|---|---|
| Cu | 63.31 | 63.43 | 0.19 |
| Ni | 14.87 | 15.28 | 2.76 |
| Zn | 20.81 | 20.14 | 3.22 |
| Mn | 0.32 | 0.34 | 6.25 |
| Fe | 0.47 | 0.56 | 19.15 |
| Si | 0.146 | 0.25 | 6.67 |
| Mg | 0.038 | — | — |
| Pt | 0.019 | — | — |
| As | 0.0098 | — | — |
| Sb | 0.0020 | — | — |
| Bi | 0.0019 | — | — |
| P | 0.0048 | — | — |

—, Compositions cannot be detected in the analytical instrument. Experimental condition: accelerate voltage, 25 kev; tilt angle, 0°; take-off angle, 38°.

for the corresponding relationship between the intensity distribution and the image.

The intensity distribution analysis is also for quasidigital mapping because it was not necessary to control the scanning mode of the electron beam and the digitalization of the electron image through image sampling.

### Results and examples

A typical analytical example is shown in figure 3. It is a graphical user interface (GUI) serving as a panel for control and display of the results of a VI in a quantitative analysis routine. The sample measured is a standard sample (National Standard GBW02104, Zn/Cu alloy). The spectra was collected and evaluated by the system. It is a test of the whole system including the spectrometer, the spectra processing and the quantitative methods. The specimen is a chip with a curve surface that is flattened with a mechanical pressure about 800 kg. The results are given in table 1 and show that the accuracy is within 5% of the certified values for most of the elements. Larger differences are found only for the elements close to the detection limit.

An example of quasidigital mapping on PC is given in figure 4, which includes line and image distribution. In the function, the mapping provides multi-elemental analysis once due to the power of computer. It can also fulfil the colour multi-elemental mapping within an image. The above works use custom-designed distribution on a PC and requires an online SEM image-analysis system to carry out the image collection.

### Conclusion

A virtual instrument of EDXS for SEM is described. All of the main components, with the exception of the Si (Li) detector and analogue signal-processing circuit, are built into a single circuit board. It was developed to replace the old EDXS in use in SEM. The system is inexpensive (total cost under $6000), and is smaller, faster and more flexible than the old one. It can be used with the new Si (Li) detector expediently. The system was used for computer-aided teaching (CAT) to accomplish the instrument development and operation.

The whole system has been running since 1999 in the SEM without any inner malfunction. Several new applications and developments based on the architecture are in development.

### Acknowledgements

The work was supported by the Department of National Science & Technology.

### References

1. Goldstein, J. I. *et al.* (eds), *Scanning Electron Microscopy and X-ray Microanalysis* (New York: Plenum, 1981).
2. Szal^oki, I., Torok, S. B., Ro, C.-U., Injuk, J. and van Grieken, R. E., *Anal. Chem.*, **72** (2000), 211R.
3. Torok, S. B., Labar, J., Injuk, J. and van Grieken, R. E., *Anal. Chem.*, **70** (1998), 495R.
4. Torok, S. B., Labar, J., Schmeling, M. and van Grieken, R. E., *Anal. Chem.*, **68** (1996), 467R.
5. Wang, C. and Gao, R. X., *IEEE Trans. Instrum. Meas.*, **49** (2000), 325.
6. Junfeng Lei, Libo Zeng, Juntang Liu and Ronggui Liu, *Development of Hardware of VI System for SEM-EDXS*. Technical Report in Group of Instrument Development, August 1999.
7. Libo Zeng, Ronggui Liu, Junfeng Lei and Zelan Zhang, *Development of Software of VI System for SEM-EDXS*. Technical Report in Group of Instrument Development, January 2000.

